# Molecular epidemiology, antimicrobial susceptibilities and resistance mechanisms of *Streptococcus pyogenes* isolates resistant to erythromycin and tetracycline in Spain (1994–2006)

**DOI:** 10.1186/1471-2180-12-215

**Published:** 2012-09-21

**Authors:** Virginia Rubio-López, Sylvia Valdezate, David Álvarez, Pilar Villalón, María José Medina, Celia Salcedo, Juan-Antonio Sáez-Nieto

**Affiliations:** 1 Servicio de Bacteriología, Centro Nacional de Microbiología, Instituto de Salud Carlos III, Majadahonda, Crta. Majadahonda-Pozuelo km 2, Madrid, 28220, Spain

**Keywords:** GAS, *emm* gene, PFGE, Macrolide resistance, Tetracycline resistance

## Abstract

**Background:**

Group A *Streptococcus* (GAS) causes human diseases ranging in severity from uncomplicated pharyngitis to life-threatening necrotizing fasciitis and shows high rates of macrolide resistance in several countries. Our goal is to identify antimicrobial resistance in Spanish GAS isolates collected between 1994 and 2006 and to determine the molecular epidemiology (*emm*/T typing and PFGE) and resistance mechanisms of those resistant to erythromycin and tetracycline.

**Results:**

Two hundred ninety-five out of 898 isolates (32.8%) were erythromycin resistant, with the predominance of *emm*4T4, *emm*75T25, and *emm*28T28, accounting the 67.1% of the 21 *emm*/T types. Spread of *emm*4T4, *emm*75T25 and *emm*28T28 resistant clones caused high rates of macrolide resistance. The distribution of the phenotypes was M (76.9%), cMLS_B_ (20.3%), iMLS_B_ (2.7%) with the involvement of the erythromycin resistance genes *mef*(A) (89.5%), *msr*(D) (81.7%), *erm*(B) (37.3%) and *erm*(A) (35.9%).

Sixty-one isolates were tetracycline resistant, with the main representation of the *emm*77T28 among 20 *emm*/T types. To note, the combination of *tet*(M) and *tet*(O) tetracycline resistance genes were similar to *tet*(M) alone reaching values close to 40%. Resistance to both antibiotics was detected in 19 isolates of 7 *emm*/T types, being *emm*11T11 and the cMLS_B_ phenotype the most frequent ones. *erm*(B) and *tet*(M) were present in almost all the strains, while *erm*(A), *mef*(A), *msr*(D) and *tet*(O) appeared in less than half of them.

**Conclusions:**

Spanish GAS were highly resistant to macrolides meanwhile showed minor resistance rate to tetracycline. A remarkable correlation between antimicrobial resistance and *emm*/T type was noticed. Clonal spread of *emm*4T4, *emm*75T25 and *emm*28T28 was the main responsable for macrolide resistance where as that *emm*77T28 clones were it to tetraclycline resistance. A wide variety of macrolide resistance genes were responsible for three macrolide resistance phenotypes.

## Background

Group A *Streptococcus* (GAS) causes a broad spectrum of illness in humans, ranging from pharyngitis to severe systemic diseases. A resurgence in serious GAS infections, such as rheumatic fever, and invasive diseases, such as bacteraemia, necrotising fasciitis, septic arthritis, sepsis, pneumonia and streptococcal toxic shock syndrome, has been observed since the mid 1980s. Indeed, these have become an important cause of morbidity and mortality all over the world [1].

Penicillin is the first choice treatment. Macrolides and tetracyclines are the most common alternative antibiotics used with penicillin-allergic patients or when first line therapy fails. Increases in macrolide resistance have been reported from many countries, being in Europe, very common in the Mediterranean countries [2,3].

Streptococci have two main mechanisms of macrolide resistance: target site modification and macrolide efflux systems. The first is achieved through a family of enzymes (rRNA methylases) that methylate an adenine residue (A2058) of the 23S rRNA V domain. This leads to a conformational change that reduces the binding of macrolides, lincosamide and streptogramin B to ribosomes, conferring co-resistance to these antibiotics (the MLS_B_ phenotype). The MLS_B_ phenotype may be expressed constitutively (cMLS_B_) or inducibly (iMLS_B_). These methylases are encoded by *erm* (erythromycin ribosome methylation) genes, with the *erm*(B) and *erm*(A) the most common [3]. In the second mechanism (the efflux system), transport proteins pump C_14_ and C_15_ macrolides out of the cell (M phenotype). The M phenotype is associated with the presence of the *mef*(A) and *msr*(D) genes, which code for the transmembrane and ATP-binding domains of this pump respectively [4].

Less information is available on the characteristics of tetracycline resistance mechanisms. In streptococci, resistance to tetracycline is conferred by ribosome protection genes such as *tet*(M) and *tet*(O) and by efflux pumps encoded by the *tet*(K) or *tet*(L) genes, although these last genes are relatively rare [4].

The prevalence of antimicrobial resistance is due to several circulating clones associated with certain *emm* types. The aim of the present study was to identify antimicrobial resistance in Spanish group A *Streptococcus* (GAS) isolates and to determine the molecular epidemiology (*emm*/T typing and PFGE) and resistance mechanisms of those resistant to erythromycin and tetracycline. This study is focused on Spanish GAS population collected from a wide spectrum of clinical backgrounds and not only from carriers as occurs for other studies. The long term studied period (13 years) and the different geographical origin may allow us to obtain an approach more real to susceptibility, phenotypes, genotypes, *emm*-types and PFGE profiles distribution in Spain.

## Results

### Overall GAS susceptibility rates

All 898 Spanish GAS isolates showed susceptibility to penicillin and vancomycin. In addition, a 32.8% (295 isolates) rate of resistance to erythromycin was seen, along with 6.5% (59) resistance to clindamycin, 6.8% (61) resistance to tetracycline, and 0.3% (3) resistance to rifampin.

### Macrolide resistance phenotypes and genotypes

Two hundred ninety five (32.8%) erythromycin resistant isolates were detected among the 898 GAS isolates gathered over the 13-year collection period. The M phenotype was clearly predominant (227 isolates, 76.9%), followed by the cMLS_B_ (60 isolates, 20.3%) and iMLS_B_ phenotypes (8 isolates, 2.7%) (Table 1). The isolates with the cMLS_B_ phenotype showed high-level resistance to erythromycin and clindamycin (MIC_90_ ≥256 mg/L), whereas those with the iMLS_B_ and M phenotypes showed lower erythromycin resistance values and susceptibility to clindamycin (Table 1). To highlight, the cMLS_B_ phenotype was more predominant among invasive that in non-invasive, 43.8 and 12.6%, respectively.

**Table 1:** **Distribution of phenotypes and genotypes among macrolide-resistant *S. pyogenes *isolates**

**Phenotype**	**No. isolates (%) Invasive/non-invasive**	**Antimicrobial agent(mg/L)**				**Macrolide resistance genotype**				
			**Range**	**MIC50**	**MIC90**	***erm*(B)**	***erm*(A)**	***mef*(A)**	***msr*(D)**	**None gene**
M	227 (76.9)	Erythromycin	1- ≥ 256	32	128	50	87	224	221	1
	38 / 189	Clindamycin	0.06-0.5	0.25	0.5					
cMLS_B_	60 (20.3)	Erythromycin	8- ≥ 256	≥256	≥256	57	11	36	17	2
	32 / 28	Clindamycin	1- ≥ 256	≥256	≥256					
iMLS_B_	8 (2.7)	Erythromycin	2- ≥ 256	16	32	3	8	4	3	0
	3 / 5	Clindamycin	0.06-0.5	0.25	0.5					
Total	295 (100)	Erythromycin	1- ≥ 256	64	256	110	106	264	241	3
	73 /222	Clindamycin	0.06-0.5	0.25	256					

In the present work, the *mef*(A) (89.5%) and *msr*(D) (81.7%) genes were the most prevalent macrolide resistance determinants. *erm*(B) and *erm*(A) were observed in just 37.3% and 35.9% of isolates respectively (Table 1). Fourteen macrolide resistance genotypes were identified among the 295 erythromycin-resistant isolates (Table 2), with *msr*(D)/*mef*(A) (38%) and *msr*(D)/*mef*(A)/*erm*(A)(19.7%) the two most common combination. Both genotypes were associated with the M phenotype.

**Table 2:** **Macrolide resistance genotypes of 295 isolates of erythromycin-resistant *S. pyogenes*, indicating the phenotypes and *emm*/T types detected**

**Macrolide resistance genotype**	**No. of isolates**	**Phenotype**^**a**^			***emm*/T types**^**a**^
	**(%)**	**cMLS**_**B**_	**iMLS**_**B**_	**M**	
*erm(B)*	14 (4.7)	14	-	-	emm6T6 (1^b^), emm11T11 (5^b^)
					emm28T28 (6^c^), emm71TNT (1)
					emm78T11 (1)
*erm(B)/erm(A)*	1 (0.3)	1	-	-	emm12T12
*erm(B)/ msr(D)*	5 (1.7)	5	-	-	emm11T11 (1^b^), emm28T28 (3)
					emm88T28 (1)
*erm(B)/mef(A)*	21 (7.1)	20	-	1	emm4T4 (1), emm28T28 (18)
					emm28TNT(1), emm75T25 (1)
*erm(B)/ msr(D)/mef(A)*	33 (11.2)	8	-	25	emm1T1 (1), emm2T2 (1)
					emm4T4 (14), emm6T6 (2)
					emm11T11 (2^b^), emm12T12 (4)
					emm28T28 (4), emm75T25 (4)
					emm84T25 (1)
*erm(B)/ msr(D)/ erm(A)*	2 (0.7)	2	-	-	emm11T11 (2^b^)
*erm(B)/ erm(A)/mef(A)*	7 (2.4)	5	2	-	emm11T11 (1^b^), emm28T28 (4)
					emm77T28 (1^b^), emm83TNT (1^b^)
*erm(B)/ msr(D)/mef(A)/ erm(A)*	27 (9.2)	2	1	24	emm1T1 (1), emm4T4 (3)
					emm11T11 (1), emm12T12 (3)
					emm75T25 (14),emm81TB3264(1)
					emm84T25 (4)
*erm(A)/mef(A)*	6 (2.0)	1	1	4	emm2T2 (3), emm28T28 (2)
					emm77T28 (1^b^)
*erm(A)*	2 (0.7)	-	2	-	emm3T3/13 (1), emm22T12 (1)
*erm(A)/ msr(D)*	3 (1.0)	-	2	1	emm22T12 (2), emm75T25 (1)
*msr(D)*	1 (0.3)	-	-	1	emm3T3 (1)
*msr(D)/mef(A)*	112 (38.0)	-	-	112	emm1T1 (6), emm4T4 (62),
					emm6T6 (26), emm12T12 (10)
					emm28T28 (1), emm75T25 (6)
					emm84T25 (1)
*msr(D)/mef(A)/ erm(A)*	58 (19.7)	-	-	58	emm1T1 (1), emm4T4 (36)
					emm12T12 (2), emm12TNT (1)
					emm44T5/27/44 (1), emm75T25 (17)
None gene	3 (1.0)	2	-	1	emm1T1 (1), emm28T28 (1)
					emm28TNT (1)
Total	295 (100)	60	8	227	

^a^No. of GAS isolates. ^b^All isolates were also resistant to tetracycline, showing co-resistance to both erythromycin and tetracycline. ^c^One isolate showed co-resistance to both erythromycin and tetracycline.

### Tetracycline resistance phenotypes and genotypes

Tetracycline-resistant phenotype was observed in 61 isolates (6.8%), showed MICs ranging from 8 to 64 mg/L (MIC_50_ 16 mg/L, MIC_90_ 32 mg/L) with a genotype distribution of *tet*(M)/*tet*(O) (42.6%), *tet*(M) (39.3%) and *tet*(O) (18.0%).

### Erythromycin and tetracycline co-resistance

Co-resistance was detected in 19 isolates (2.1%). The erythromycin MIC was >256 mg/L for 18 isolates and just 32 mg/L for one isolate. The clindamycin MICs were also high at >256 mg/L for 14 of the 19 isolates. All isolates except one (iMLSB) had the cMLSB macrolide resistance phenotype. The resistance genes detected were *erm*(B) (94.7%), *erm*(A) (42.1%), *mef*(A) (47.4%), *msr*(D) (36.8%), *tet*(M) (100.0%) and *tet*(O) (36.8%), with *tet*(M) the only tetracycline resistance determinant in 13 isolates, while in 6 it was simultaneously detected with *tet*(O) (Table 3).

**Table 3:** **Distribution of * emm/*T types and resistance genes in *S. pyogenes *resistant to erythromycin and tetracycline with respect to the overall Spanish GAS population**

***emm***	**T**	**No. of isolates/Total**	***erm*(B)**	***erm*(A)**	***mef*(A)**	***msr*(D)**	***tet*(M)**	***tet*(O)**
**Erythromycin-resistant (n = 276)**								
1	1	9/129	1	2	8	8		
2	2	4/41	1	3	4	1		
3	3	1/26	0	0	0	1		
3	3/13	1/5	0	1	0	0		
4	4	116/137	18	39	116	115		
6	6	28/67	2	0	28	28		
11	11	1/24	1	1	1	1		
12	12	19/68	7	5	18	18		
12	NT	1/1	0	1	1	1		
22	12	3/13	0	3	0	2		
28	28	37/78	33	5	28	8		
28	NT	2/2	1	0	1	0		
44	5/27/44	1/20	0	1	1	1		
71	NT	1/1	1	0	0	0		
75	25	43/46	19	32	42	42		
78	11	1/30	1	0	0	0		
81	B3264	1/1	1	1	1	1		
84	25	6/6	5	4	6	6		
88	28	1/1	1	0	0	1		
Total		276/898	92	98	255	234		
**Tetracycline-resistant (n = 42)**								
6	6	1/67					1	0
11	11	1/24					1	0
22	12	2/13					2	2
31/13	NT	1/1					1	1
33	3/13	1/1					1	0
36	NT	1/1					1	0
50	NT	1/2					1	1
58	NT	1/6					1	0
60	28	4/5					4	1
73	13	2/11					2	2
77	13	1/7					1	0
77	14/49	1/1					1	0
77	28	21/23					11	20
78	11	1/30					1	0
87	28	2/50					2	2
NT	NT	1/1					0	1
Total		42/898					31	30
**Erythromycin -Tetracycline-resistant (n = 19)**								
1	1	1/129	1	0	1	1	1	1
6	6	1/67	1	0	0	0	1	1
11	11	11/24	11	3	3	5	11	3
12	12	1/68	1	1	1	1	1	0
28	28	2/78	2	1	1	0	2	0
77	28	2/23	1	2	2	0	2	2
83	NT	1/1	1	1	1	0	1	0
Total		19/898	18	8	9	7	19	7

### T- serotypes and *emm* types (*emm*/T types) distribution

Twenty one *emm/*T types were observed in the erythromycin-resistant population (295) (Table 3), the 6 most common being *emm*4T4 (39.3%), *emm*75T25 (14.6%), *emm*28T28 (13.2%), *emm*6T6 (9.8%), *emm*12T12 (6.8%) and *emm*11T11 (4.1%) which represented 87.8% of the erythromycin-resistant isolates. High macrolide resistance rates were associated with the above *emm*/T types: *emm*75T25 (93.5%), *emm*4T4 (84.7%), *emm*11T11 (50%), *emm*28T28 (50%), *emm*6T6 (43.3%) and *emm*12T12 (29.4%).

In the present tetracycline-resistant population (61), 20 different *emm*/T types were identified (Table 3). *emm*77T28 (37.3%) was the main *emm*/T type associated with tetracycline resistance; all *emm*77T28 isolates detected over the 13 years of the study were resistant to this antibiotic.

In the erythromycin- and tetracycline-resistant population population (19), 7 *emm*/T types were observed, the majority being *emm*11T11 (57.8%) (Table 3); indeed, 45.8% of all *emm*11T11 recovered from the initial GAS population (898) were co-resistant.

The correlation between the different *emm/*T types and macrolide resistance genotypes is shown in Table 2. The *mef*(A)/*msr*(D) gene complex was the most common in almost all *emm*/T types, either alone or in combination with other genes. The *mef*(A)/*msr*(D) genotype was the most common in the *emm*1T1 (6/10), *emm4*T4 (62/116), *emm*6T6 (26/29) and *emm*12T12 (10/20) types. The *msr*(D)/*mef*(A)/*erm*(A)(36/116) was the most common genotype among the *emm*4T4 (36/116) and *emm*75T25 (17/43) types.

### PFGE typing

**Figure 1:**
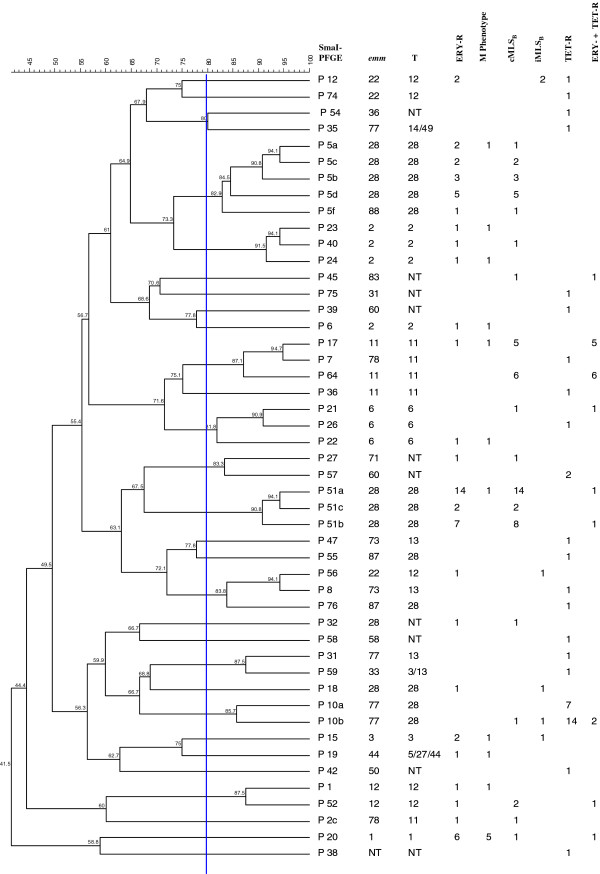
***Sma*I-pulsotypes, *emm/*T and phenotypes of erythromycin- and/or tetracycline-resistant* S. pyogenes.***

In the erythromycin-resistant population (295 isolates), 79 (26.8%) *SmaI*-restricted and 216 (73.2%) *SmaI*-non-restricted isolates were identified. *SmaI*-restricted isolates generated 30 pulsotypes with a similarity range of 38.8% to 94.7% (Figure 1). Their distribution by phenotype was: M (11 isolates), cMLS_B_ (58) and iMLS_B_ (6).

**Figure 2:**
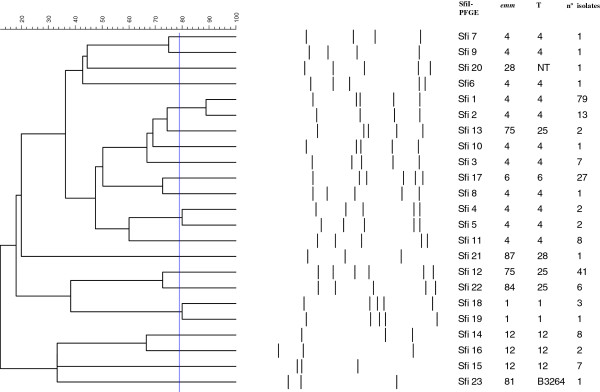
**PFGE-pulsotype generated with *Sfi*I in 216 *Sma*I-unrestricted isolates resistant only to erythromycin, indicating emm-T type.**

The 216 *SmaI*-non-restricted isolates (Table 4) were typed with *Sfi*I, generating 22 pulsotypes with a similarity range of 12.2% to 88.9% (Figure 2). The M phenotype (212 isolates) predominated over the cMLS_B_ (2) and iMLS_B_ (2) phenotypes. In addition, 11 different *emm*/T types were detected (Table 4) among 216 *SmaI*-non-restricted isolates, the most common being *emm*4T4 and *emm*75T25. All *emm*4T4 and all *emm*75T25 erythromycin-resistant isolates but one were *Sma*I non-restricted and had the M phenotype; together these accounted for 53.9% of the macrolide-resistant isolates in our study.

**Table 4:** **Distribution of *emm*/T types, phenotypes and genotypes of erythromycin-resistant *Sma*I-non-restricted isolates**

***emm***	**T**	**Phenotype**	**No. of isolates**	**Genotypes (no. of isolates)**			
				***erm*(B)**	***erm*(A)**	***msr*(D)**	***mef*(A)**
1	1	M	4	1	2	4	4
12	12	M	16	4	2	16	16
12	NT	M	1	0	1	1	1
28	28	iMLS_B_	1	1	1	0	1
4	4	M	116	18	39	115	116
6	6	M	27	1	0	27	27
75	25	M	42	18	32	42	41
75	25	cMLS_B_	1	1	0	0	1
81	B3264	iMLS_B_	1	1	1	1	1
84	25	M	6	1	4	6	6
28	NT	cMLS_B_	1	1	0	0	1
Total			82	216			

In the case of tetracycline-resistant isolates, all were *SmaI*-restricted, generating 30 pulsotypes with a similarity range of 42.16 to 100.0% (Figure 1). The *Sma*10a *emm*77T28 and *Sma*64 *emm*11T11 pulsotypes may be associated with tetracycline resistance since 100% of these isolates were resistant to this antibiotic. All co-resistant (erythromycin and tetracycline) isolates were *Sma*I-restricted.

## Discussion

Several reports show that GAS resistance to macrolides and tetracyclines are high some countries such Spain and continue to increase; indeed, they have become clinically problematic.

In Europe, the most northerly countries (with the exception of Finland) have reported low levels of resistance (<4%) [5] while strong resistance has been reported from Mediterranean countries such as Italy (22,6%), France (22.4%), Greece (24.0%), Spain (21.3%) and Portugal (26.6%) [6-10]. This values contrast with those of Israel (1.8%) and Iran (0.2%) [11,12].

In our study, 32.8% of isolates showed resistance to macrolides. Efflux pumps (M phenotype) are one of the major mechanisms conferring resistance to macrolide antibiotics, and streptococci making use of this system have been commonly reported from European countries, Argentina, the USA and Canada [5,13-15]. The M phenotype has been identified as predominant in several Spanish studies, reaching a rate of 95.6% in a multicentre study undertaken in 1998 or 64.5% in an extensive national multicenter surveillance study in 2006–2007 [16,17]. In the present population, the efflux system was also the main macrolide resistance mechanism seen, being manifested by 76.9% of isolates.

cMLS_B_ phenotype, another common phenotype reported in Europe [18], was displaced by the M phenotype in several European countries from 1990 [10,19]. In our study, cMLS_B_ phenotype was the second most commonly encountered (20.3%) like SAUCE project carried out in 2006–2007 [17]. In this last report, flutuations in the rates of resistance to macrolides are observed (1996–1997: 26.7%; 1998–1999: 20.4%; 2001–2002: 24.3; 2006–2007: 19%) meanwhile there is an increasing trend in the prevalence of MLS_B_ phenotype from 14% in 2001–2002 to 35.5% in 2006–2007 [17].

Among Spanish isolates of this work, iMLS_B_ phenotype was minority (2.7%) in contrast to Norway (75%) (1993–2002) or Bulgaria (57.7%) (1993 – 2002) where it was reported the most prevalent phenotype [5].

A gene-phenotype correlation previously described was also noticed [3,9]. *mef*(A) and *erm*(B) were predominant in isolates with the M and cMLS_B_ phenotype respectively, whereas all isolates with the iMLS_B_ phenotype harboured the *erm*(A) gene.

The *mef*(A) gene responsible for the M phenotype was detected in all but three of the present Spanish isolates with that phenotype. One of these three isolates showed none of the genes studied. In the remaining two, *msr*(D) was observed alone or in combination with *erm*(A). In these last two cases, the *msr*(D) gene might be only one of the determinants responsible for the M phenotype. *msr*(D) and *mef*(A) have been placed in the same genetic element [8,20], suggesting that the proteins they encode may act as a dual efflux system. However, it has also been suggested that the *msr*(D)-encoded pump can function independently of the mef-encoded protein [20].

The *erm*(B) gene responsible for the cMLS_B_ phenotype was identified in all but three of the present isolates with this phenotype. None of genes tested could be amplified in two isolates, indicating that other resistance genes must be involved. The remaining isolate harboured *erm*(A) and *mef*(A). In this case, *erm*(A) may be responsible for the cMLS_B_ phenotype since alterations in the regulatory region of the gene have been identified that induce constitutive expression [21].

An ample macrolide resistance genes combination was identified, specifically fourteen genotypes. Interestingly, single genotypes could show one or several phenotypes, a phenomenon reported by other authors [5,10]. One of these, *erm*(B)/*msr*(D)/*mef*(A) genotype showed M and MLS_B_ phenotypes in 25 and 8 isolates respectively, while the *erm*(B)/*erm*(TR)/*msr*(D)/*mef*(A) genotype showed all three macrolide resistance phenotypes. Nowadays, this correlation between genotype and phenotype is not well understood.

In our erythromycin-resistant population (295), the 6 most common *emm*/types: *emm*4T4 (39.3%), *emm*75T25 (14.6%), *emm*28T28 (13.2%), *emm*6T6 (9.8%), *emm*12T12 (6.8%) and *emm*11T11 (4.1%) have been previously associated with macrolide resistance in numerous reports [6,10,12,14]. *emm*28 and *emm*4 have been reported the most common in Europe (2003–2004) [18], and to be responsible for an increase in erythromycin resistance among GAS in Spain, Finland and Quebec [6]. *emm*12 is the main resistant *emm* type in Germany, Greece, Italy, Portugal, Israel [10,12,13] and the second one in the United States, being surpassed only by *emm*75 [14].

Most of erythromycin-resistant isolates were *Sma-*non-restricted (73.2%) due to the presence prophage-like elements that confer the M phenotype and harbour the *mef*(A) and *msr*(D) genes. These genetic elements encode a DNA-modifying methyltransferase that acts on the *Sma*I recognition sequence and renders DNA refractory to cleavage by *Sma*I [21]. All but four of the present *Sma*I non-restricted isolates were susceptible to tetracycline and had an M phenotype. This suggests that these isolates carry *mef*(A) and *msr*(D) contained within a Tn1207.1 transposon inserted into a larger genetic element such as the Tn1207.3 or 58.8 kb chimeric element, flanked by the *comEC* gene from the Tn1207.3/Φ10394.4 family [22]. In our study, all *emm*4T4 and all *emm*75T25 erythromycin-resistant isolates but one were *Sma*I non-restricted and had the M phenotype; together these accounted for 53.9% of the Spanish macrolide-resistant isolates. Several resistant clones previously described in Spain were identified [9,10]. The *emm*4T4 *Sfi*1 (79) clone resembles to clone B described in 1999 [10]. It was the most common in the present study, indicating it to still be circulating in Spain. This clone has a wide distribution, and it has recently been identified in Finland, Greece, Italy, England and Sweden [23]. Clone C, previously identified in Spain, the United Kingdom and the United States [23] was not detected among the present isolates, although it might be related to the present clones *emm*4T4 *Sfi*4 and *emm*4T4 *Sfi*5.

The major macrolide-resistant clone *emm*75T25 *Sfi*12(41) was similar (additional band between 48.5 and 97 kb) to clone D described by Perez-Trallero et al. [10]. The *emm*6T6 *Sfi*17 and *emm*84T25 *Sfi*22 clones might be associated with resistance since they were only observed in isolates resistant to erythromycin.

Regarding tetracycline resistance, we detected values of 6.8% between 1994 and 2006, indicating there to be no trend towards increased tetracycline in Spain. However, higher rates have been found in other countries such as Israel (23.6%), Denmark (33.7%), Portugal (38.7%) or Iran (42%) [10-12].

In this study, a predominance of genotype with both genes *tet*(M) and *tet*(O) (42.6%) was observed. But no Spanish reports citing the predominance of both genes appears to exist, *tet(*M) alone is usually the most common resistance determinant followed by *tet*(O) [9].

In the present tetracycline-population, *emm*77T28 was the main *emm*/T type. *emm*77 has been previously associated with resistance to tetracycline in Israel and Europe [12]. In Italy and Norway, an *emm*77 clone has been reported that is characterised by its carrying *tet*(O) linked to *erm*(A)and being associated with the iMLS_B_ phenotype [2]. In the present study, the two co-resistant *emm*77T28 isolates showed genotypes different to those described by Palmieri et al. [2].

With regard to co-resistance, we found that all isolates (19) except one had the cMLS_B_ macrolide resistance phenotype such as Greece (Athens) and Norway [5,15]. In contrast, in Finland, iMLS_B_ isolates showing co-resistance have reached rates of 93% [19]. A correlation between the M phenotype and co-resistance has been also reported [23], but this was not detected in the present study.

Of the 19 co-resistant isolates, five carried *tet*(M)/*erm*(B) as their only resistance genes, suggesting they may carry conjugative transposons of the Tn916 family in which *erm*(B) and *tet*(M) are linked [24],whereas 13 harboured *tet*(M)/*erm*(B) associated with other resistance genes. In the remaining isolate, the *erm*(B), *mef*(A), *tet*(M) and *tet*(O) genes were all detected. *mef*(A) and *tet*(O) linkage has been previously reported in co-resistant isolates [22,25]. In the present work, *mef*(A) appeared associated with other macrolide resistance genes and linked to *tet*(M) (1 isolate) or to *tet*(M)/*tet*(O) (5). The main *emm*/T type detected in coresistant isolates was *emm*11T11 (57.8%). This *emm*/T type has previously been associated with co-resistance [9,11] with an *erm*(B)/*tet*(M) clone prevalent among Spanish MLS_B_ isolates [9]. Four isolates with this genotype were found in the present work, but we can not confirm whether they belong to the above clone.

## Conclusion

In summary, the resistance against erythromycin, single or together to tetracycline, is due to a wide combination of resistance genes in Spanish GAS. Erythromycin resistance is mainly consequence of clonal spread of *emm*4T4, *emm*75T25, both associated with M phenotype and *SmaI* non-restricted, and *emm*28T28. Whereas tetracycline resistance and coresistance is due to clonal spread of *emm*77T28 and *emm*11T11, respectively, all *Sma*I restricted.

## Methods

### Bacterial isolates

Between 1994 and 2006, 898 GAS isolates were submitted for their characterisation to the Streptococcal Reference Laboratory from 75 Hospitals and Public Health Laboratories in 32 Spanish provinces. GAS identification was confirmed by colony morphology, β-haemolysis on blood agar, a latex agglutination assay (Slidex, Streptokit, BioMerieux, Marcy-LÂ´Etoile, France), and by using the rapid ID 32 STREP kit (BioMerieux, Marcy-LÂ´Etoile, France). The erythromycin- and tetracycline-resistant isolates were selected as the study population (see section antimicrobial susceptibility tests). This population (337 isolates) was collected from a wide spectrum of clinical backgrounds, including necrotising fasciitis (3), cellulitis and other skin infections (67), streptococcal toxic shock syndrome (13), sepsis and meningitis (17), respiratory infection (5), bone infection and rheumatic fever (4), genital infection (20), otitis (12),conjunctivitis (1), scarlet fever (70) and pharyngotonsillitis (80), as well as from asymptomatic carriers (45). For the latter status, the GAS isolates were recovered from oropharyngeal swabs. A limitation of the study was due to the voluntary nature of the submission of these strains, producing a bias in the annual number.

### Antimicrobial susceptibility tests

The minimum inhibitory concentrations (MICs) of penicillin, vancomycin, erythromycin, clindamycin, tetracycline and rifampin were determined using the E-test (AB Biodisk, Solna, Sweden) following the standard method [26]. Susceptibility results were categorized according to the criteria of the Clinical and Laboratory Standards Institute [26]. The erythromycin- (MIC ≥ 1 mg/L) and tetracycline-resistant (MIC ≥ 8 mg/L) isolates were then selected as the study population. *Streptococcus pneumoniae* ATCC 49619 was used as control.

### Detection of the macrolide resistance phenotype

Erythromycin-resistant isolates were classified on the basis of their susceptibility patterns as shown by double-disk tests involving erythromycin (15 μg) and clindamycin (2 μg ) disks (Becton Dickinson Microbiology Systems, Cockeysville, MD, USA) [27]. Three phenotypes were assigned: M (erythromycin resistant and clindamycin susceptible), cMLS_B_ (constitutive erythromycin and clindamycin resistant), and iMLS_B_ (erythromycin resistant and clindamycin inducible). Blunting of the clindamycin inhibition zone near to the erythromycin disk indicated an iMLS_B_ phenotype, whereas susceptibility to clindamycin with no blunting indicated the M phenotype.

### Detection of erythromycin and tetracycline resistance genes

All erythromycin-resistant isolates were screened by PCR for the erythromycin resistance genes *erm*(B) [28], *erm*(A) [3], *mef*(A) [4], and *msr*(D) [29]. Tetracycline-resistant isolates were tested for the tetracycline resistance genes *tet*(M) and *tet*(O) [4]. PCR assays were carried out according to previously described conditions for each individual primer pairs.

### T-serotype and *emm* type (*emm*/T types)

The T-serotype was determined by slide agglutination using type-specific antisera (Seiken-Oxoid, Cambridge, UK). *emm* sequencing was performed according to the protocol of the CDC International Streptococcal Reference Laboratory (http://www.cdc.gov/ncidod/biotech/strep/protocols.htlm).

### Pulsed field gel electrophoresis (PFGE) analysis

PFGE was performed as previously described [30] with slight modifications. Chromosomal DNA was digested with the *Sma*I (40U) restriction enzyme (Fermentas, Vilnius, Lithuania) for 4 h at 30°C and the electrophoresis conditions were 22 h with an 0.5 to 40s switch time ramp at a 120° angle and 6 V/cm. *Sma*I non-restricted isolates were typed by PFGE using the *SfiI* restriction enzyme (Fermentas, Vilnius, Lithuania) under previously described conditions [31]. The PFGE profiles were analysed using InfoQuest FP software v.4.5 (Bio-Rad Laboratories, Hercules, CA, USA), employing the UPGMA method with the Dice coefficient and a position tolerance of 1.2%. *Sma*- and *Sfi*-profiles were number-coded. For closely related *Sma-*types (1–2 bands of difference) a letter was added.

### Financial competing interest

This research was funded by an intramural predoctoral fellowship from the Carlos III Health Institute (grant number 05/0030) and the Spanish Ministry of Science and Innovation.

## Competing interests

The authors declare that they have no competing interests.

## Authors’ contributions

PV, MJM, SV, JA and VR participated in the molecular data collection and analysis. DA, CS and VR conducted the microbiological methods and analysed data. SV, JA and VR interpreted data, and drafted the manuscript. SV and JA were involved in critically revising the manuscript. All authors read and approved the final manuscript.
